# Prevalence and access to disability inclusive social protection services in Somalia: status, gaps, and policy implications (a secondary re-analysis of survey data)

**DOI:** 10.11604/pamj.2025.52.153.44405

**Published:** 2025-12-09

**Authors:** Gilbert Koome, Mohammed Hassan, Naima Ibrahim, Fredrick Odinga, Leylah Abdullahi, Lisa Were

**Affiliations:** 1 Horn Population Research and Development, East, and Horn of Africa Region, Nairobi, Kenya

**Keywords:** Disability, social protection, inclusion, policy, frameworks

## Abstract

**Introduction:**

persons with disabilities are among the most marginalised groups in Somali society. This paper provides evidence-based information on the prevalence, characteristics of disability, and barriers to disability-inclusive social protection services in Somalia.

**Methods:**

the paper is based on a secondary re-examination of data from the Somalia Integrated Household Budget Survey (2022) of 7,212 households.

**Results:**

disability prevalence in Somalia was estimated at 16.2% with the most common being physical impairment, affecting 9.8% of the population. The at-risk groups were the elderly population and children. Regionally, disability prevalence was highest in Banadir State: 21.5%. Enrollment rates in social protection schemes were relatively low: 15% of surveyed households. Access to disability-specific services and specialists was generally limited across all disability types, with vision screening being the most accessed service and learning screening being the least accessed service. Access to assistive devices is also very low, with only 4% of people with disabilities who need them receiving them. Access to social protection delivery systems, such as phone, internet, and bank accounts, was also low <20% for each service.

**Conclusion:**

the study reveals a high prevalence of disability and low access to social protection and disability-specific services in Somalia, especially among women, rural residents, older people, and those affected by conflict. The study indicates a need for comprehensive disability data collection that reflects the diverse needs and barriers to reinforcing disability-inclusive policy frameworks faced by persons with disabilities in Somalia.

## Introduction

Somalia is a federal republic composed of five member States: Galmudug, Hirshabelle, Jubaland, Puntland, and Southwest State. It also includes the self-declared but unrecognized Somalia and the capital city of Mogadishu, which hosts the Federal Government of Somalia. Somalia is one of the poorest and most fragile countries in the world, ranked 194 out of 189 countries in the Human Development Index (HDI) in 2020 [1]. According to the World Bank, 69% of the population lives below the poverty line of $1.90 per day, and 51% lives in extreme poverty of less than $1 per day [2]. The country has been affected by decades of conflict, instability, drought, famine, and displacement, which have undermined its social and economic development. An estimated 5.9 million people, or 38% of the population, need humanitarian assistance as of November 2020, and 2.6 million are internally displaced. Somalia is also vulnerable to the effects of climate change, such as floods, cyclones, and desert locust infestations, which threaten food security and livelihoods. Disability inclusion is a critical aspect of social protection policies and programs. Approximately one billion people, constituting 15% of the world´s population, experience some form of disability (World Health Organization (WHO) and World Bank, 2011) (3). These individuals often face adverse socioeconomic outcomes compared to those without disabilities. The prevalence of disability is higher in developing countries, where barriers to inclusion persist. The World Bank (2019) defines disability as a condition that limits an individual´s ability to participate fully in social, economic, and cultural life. The prevalence of disability is higher in developing countries, where 80% of persons with disabilities live [3]. In Africa, the disability prevalence rate is estimated at 15%, which is above the global average of 10% [4].

The main causes of disability in Africa include malnutrition, infectious diseases, conflicts, and environmental hazards. Persons with disabilities in developing countries face multiple challenges, such as a lack of access to health care, education, employment, social services, and legal protection [3]. Social protection encompasses policies and programs aimed at reducing poverty, vulnerability, and inequality. It includes measures such as cash transfers, health insurance, and employment support. The United Nations Convention on the Rights of Persons with Disabilities (CRPD), ratified by 185 countries, emphasizes the full integration of persons with disabilities into societies. It underscores the importance of international development in addressing their rights [5]. The 2030 Agenda for Sustainable Development explicitly states that disability should not be a reason for lack of access to development programming and human rights realization [6]. The Sustainable Development Goals (SDGs) framework includes targets related to persons with disabilities. The COVID-19 pandemic has aggravated the challenges facing persons with disabilities across sectors such as health, education, and transportation [7]. For instance, the pandemic has increased the risk of infection, morbidity, and mortality for persons with disabilities, especially those with underlying health conditions or living in institutional settings [8]. The pandemic has also disrupted the provision of essential services and support for persons with disabilities, such as rehabilitation, assistive technology, personal assistance, and mental health care [9]. Lack of accessible information and services due to systemic, process and structural reasons such as weak representation, lack of disability-sensitive service models, low awareness, and social support services, exacerbates their vulnerability [10]. Approximately one-third of countries have adopted specific measures for persons with disabilities during the pandemic [11].

These measures include extra cash transfers, adapted delivery methods, home delivery of essential items, and helplines for information and support. However, only 27.8% of persons with severe disabilities worldwide receive disability benefits [12]. Regional disparities exist, with almost universal coverage in Eastern Europe but only 9.4% coverage in Asia and the Pacific. Low coverage is linked to narrow and inaccessible methods of disability assessment and means testing. To address this gap, Governments, such as the Federal Government of Somalia, can enhance disability inclusion by mainstreaming it into social protection policies and ensuring accessible services. Training programs for social workers and service providers have also been shown to improve disability awareness and responsiveness, while partnerships between governments, civil society, and international organizations drive progress. However, overcoming societal prejudices and promoting positive attitudes toward disability, allocating sufficient resources to disability-inclusive social protection, and improving data collection and monitoring mechanisms to track progress are some of the challenges that need to be addressed [6]. As a result, disability inclusion in social protection responses remains a global priority. One structural initiative to strengthening disability-inclusive social protection responses in Somalia is the enactment of the National Social Protection Policy in 2019, which recognizes persons with disabilities as a key target group and commits to providing them with adequate and regular cash transfers and other social services. The policy also aims to mainstream disability across all sectors and promote their participation and empowerment [13]. The main social protection programs in Somalia are cash transfers, food vouchers, and school feeding, which are mostly funded by external donors and implemented by humanitarian actors. Nevertheless, the state of disability-inclusive social protection service in Somalia is not documented, impacting targeted policy and service advocacy. Objective: This study´s objective was to document the state of social-protection inclusiveness in Somalia. This evidence is expected to inform the creation of more equitable and inclusive social protection systems that benefit all individuals, regardless of their abilities.

## Methods

**Study design:** the study was a secondary re-analysis of data from the Somalia Integrated Household Budget Survey (SIHBS) conducted in 2022 across the country [14].

**Study setting:** the survey included Puntland, Galmudug, Hirshabelle, Southwest State, Jubaland, and Banadir regions of Somalia, which is one of the countries located within the Horn of Africa. The SIHBS was a survey that reflected the national population based on a sample of 7,212 households from rural, urban, and nomadic households in Somalia. The survey used the same sample frame as the Somalia Health Demographic Survey (SHDS) 2020, which covered urban, rural, and nomadic strata and Internally Displaced Persons (IDPs).

**Participants:** the main target for the interview was the Head of Household (HoH) and/or the spouse of the HoH. If both were absent, another member was picked, provided that he/she was a HH member aged 16 and above, was not a dependent, and knew about the composition, expenditures and consumptions of the household.

**Variables:** independent variables included population demographics (such as age, gender, geographical location, and region), socioeconomic status and household composition. The outcome variables were disability, defined as the status and type (visual, physical or hearing impairment) and access to basic services and social protection (social protection services and social protection delivery systems). Additionally, perceived fairness of social protection, availability and suitability of feedback mechanisms.

**Data source and measurement:** the survey used a questionnaire as the data collection tool; it had two modules: (i) a demographic module that gathered information on the population, such as demographics and disability and ii) a household module that gathered information on access to basic services. Disability assessment applied functional measurement using the Washington Group short set of questions on disability. The survey questionnaire and listing form were programmed with the Survey solutions software to enable computer-assisted personal interviewing (CAPI) using tablets.

**Bias:** the mobile script was tested and checked to make sure that all the questions, skip logics, and iterations were correctly captured. In addition, the instruments were piloted in English. The pilot helped the survey team to check for language clarity, questionnaire structure and question flow, skip logic and interview duration. The data collection followed strict quality control procedures, which were applied at different stages of the fieldwork. Quality assurance procedures included supervised interviews, spot-checks, back-checks, and automated high-frequency checks of the data collected. Each region had a separate quality control team that reported directly to the project manager. Supervisors monitored 10-15% of all interviews done by each interviewer; this monitoring was more intense during the first days of data collection to assess and improve interviewer performance by identifying errors. In addition, back-checking was done on 5% of the interviews.

**Study size:** the SIHBS sample of 7,212 households was chosen from 601 enumeration areas (EAs) that were spread across Somalia; including all the five federal member States of Somalia, namely: Puntland, Galmudug, Hirshabelle, Southwest State, and Jubaland as well as Benadir Region Administration. About 35 EAs were sampled in each of the 17 covered regions, with 12 HHs interviewed per EA, adding up to about 420 HHs per region [14].

**Statistical methods:** the data was analyzed using descriptive statistics like percentages and frequencies to show the features of the population and the rates of different disabilities and access to social protection services. The findings were displayed in tables and graphs to show the variation and trends of disability and access to social protection services among population groups, regions, and urban-rural locations.

## Results

**Participants:** a total of 7,212 households were included, chosen from 601 enumeration areas (EAs). The average household size is 6.7 persons nationally. The largest population age group is 5-9 years 18.9%, with 0.7% of the population being 80+ years. Half (50.5%) of the population above 15 years were married and 37.9% were unmarried.

**Descriptive and outcome data:** the disability prevalence in Somalia is estimated at 16.2%. The most common type of disability was physical (walking) impairment, affecting 9.8% of the population, followed by visual impairment (6% and 4%), and hearing impairment (5.3%). The types of disability reported varied across different demographic groups. For example, visual impairment was more common among females, the elderly, and those living in rural areas or IDP camps, while hearing impairment was more common among males and those living in urban areas.

**Figure 1:**
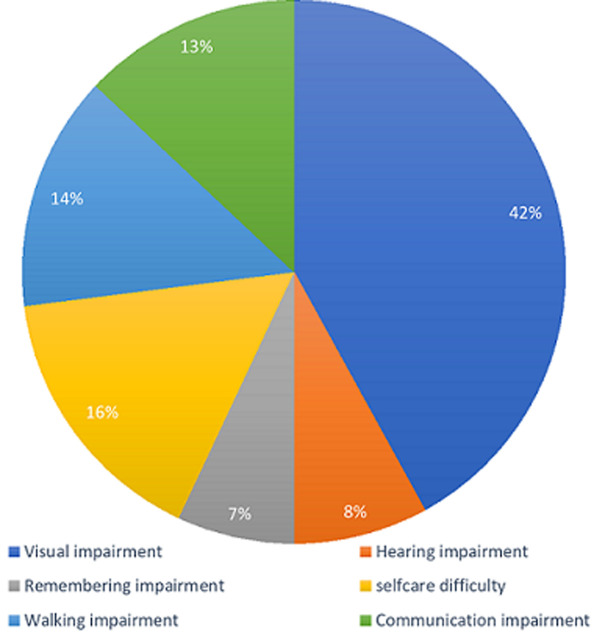
percentage of registered PwDs receiving social protection cash transfers

**Figure 2:**
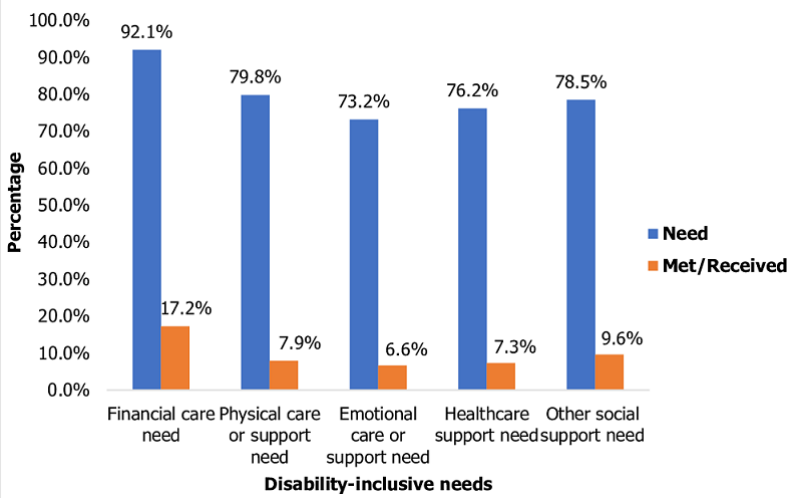
need and access to disability-inclusive/sensitive services

Regionally, the disability prevalence was highest in Banadir State (21.5%) and in terms of residence, disability prevalence was higher in rural areas (17.1%). Table 1 shows that the most at-risk groups based on disability prevalence are the elderly population (65+), who had the highest prevalence of walking difficulty and multiple disabilities, and children with disabilities (0-14), who had relatively high prevalence rates of self-care difficulty and communication impairment. As shown in Figure 1, access to general social support services is also low across all disability types, with a significant gap between the need and the receipt of services such as financial support, physical care or support, emotional care or support, healthcare support, and other social support. As shown in Figure 2, most of the assistance received by persons with disabilities comes from informal networks and external organizations, while awareness of the government's role in social protection remains very low at the community level. Access to social protection delivery systems, such as phone, internet, and bank accounts, was low among persons with disabilities, with percentages below 20% for each service (Table 2). The perceived fairness of the selection criteria for social protection beneficiaries is low. For instance, about 30% of respondents said they knew other eligible persons with disabilities who were not receiving benefits. About 8% of households perceived some beneficiaries as unfairly chosen. The availability and suitability of feedback and grievance redress mechanisms, which include reporting via phone and community leaders, is also low. About 35% of respondents express dissatisfaction with the current system and cite reasons such as lack of trust, fear of victimization, and lack of awareness on where and how to report concerns.

**Table 1:** most at risk PWD groups

PWD group	Visual	Hearing	Walking	Remembering	Self-care	Communication
Children with Disabilities 0-9 years	1.1%	1.2%	3.0%	3.0%	3.6%	4.8%
Adolescent youth with disability 10 -19 years	0.8%	0.7%	1.0%	0.7%	0.6%	2.1%
Elderly with disabilities - 65+	35.2%	23.5%	38.0%	18.7%	19.3%	11.5%
PwDs in urban areas	2.8%	2.0%	3.5%	2.6%	2.7%	3.4%
PwDs in rural areas	2.8%	1.8%	4.0%	1.9%	2.1%	3.8%
Nomadic PwDs	2.1%	1.5%	3.1%	1.8%	1.6%	2.5%

PwDs: persons with disabilities

**Table 2:** persons with disabilities’ access to phone, internet, and bank accounts

Impairment	Phone access	Access to internet	Bank account
Visual impairment	4.1%	2.2%	4.9%
Hearing impairment	2.2%	1.1%	2.1%
Walking impairment	4.1%	1.8%	3.3%
Remembering impairment	1.5%	0.8%	1.5%
Self-care	1.3%	0.8%	1.1%
Communication impairment	1.8%	0.9%	1.5%
**Total**	15.1%	7.6%	14.4%

## Discussion

**Key results:** the overall disability prevalence rate of 16.2% in Somalia is higher than the global average of 15%, according to the WHO report on disability [3]. This shows that Somalia faces a significant burden of disability that can be attributed to various factors, such as conflict, poverty, malnutrition, disease, lack of health care, environmental hazards and cultural practices. It is important to note that the disability prevalence rate in Somalia also varies depending on the source of data and the methodology used. For instance, the United Nations Population Fund (UNFPA) estimated the disability prevalence in Somalia at 12.6% in 2020, based on the Washington Group Short Set of Questions on Disability (WGSSQ), which is a widely used tool for disability assessment in surveys and censuses [15]. The difference between the HHDI and the UNFPA estimates may be explained by the different definitions and measurements of disability used by the two sources. The HHDI survey used a functional approach to disability, focusing on the difficulties individuals experience in performing basic activities, such as seeing, hearing, walking, communicating, or self-care. The WGSSQ, on the other hand, uses a more comprehensive approach that includes not only functional difficulties but also participation restrictions and environmental barriers that affect the quality of life of persons with disabilities. Therefore, the WGSSQ may capture a wider range of disabilities and their impacts than the HHDI survey, which may result in lower prevalence rates. Moreover, the WGSSQ has been tested and validated in various contexts and countries, while the HHDI survey used a custom-made questionnaire that may not have the same reliability and validity. Therefore, it is important to consider the methodological differences, and limitations when comparing and interpreting disability prevalence rates from these different sources.

The study established that the types of disability in Somalia are predominantly physical (walking) impairment, visual impairment, and hearing impairment, which account for more than 80% of the total disability cases. These types of disability are commonly associated with ageing, chronic diseases, injuries, and infections, which are prevalent in Somalia due to the low levels of health care access and quality, as well as the protracted conflict and violence that have affected the population for decades. According to the World Bank, Somalia has one of the lowest health workforce densities in the world, with only 0.4 physicians and 1.1 nurses and midwives per 10,000 population, compared to the global average of 15.6 and 28.6, respectively [16]. Moreover, Somalia suffers from frequent outbreaks of infectious diseases, such as measles, cholera, malaria, and tuberculosis, which can cause or exacerbate disability if left untreated. For example, measles can cause blindness, deafness, or brain damage in children, while tuberculosis can affect the lungs, bones, or nervous system and cause physical or sensory impairments. Furthermore, Somalia has been affected by armed conflict and civil war since 1991, which has resulted in widespread displacement, human rights violations, and casualties. According to the Armed Conflict Location and Event Data Project (ACLED), more than 8,000 civilians were killed and over 5,000 were injured by direct attacks in Somalia between 1997 and 2020 [17]. These attacks can cause traumatic injuries, such as amputations, fractures, burns, or spinal cord injuries, that can lead to permanent disability. The study also found that females have a slightly higher disability prevalence than males (17.2% vs 15.2%), and were more likely to have visual impairment, while males are more likely to have hearing impairment. This may be explained by the different roles and exposures of females and males in Somali society, as well as the different access and utilization of health care services. Females in Somalia may face greater risks of disability due to factors such as early and frequent pregnancies, female genital mutilation, domestic violence, and sexual abuse, which can affect their physical and mental health and well-being. Moreover, females in Somalia may have less access to healthcare services than males, due to socio-cultural norms, economic barriers, or security constraints, which can prevent them from seeking or receiving timely and adequate treatment for their health problems. On the other hand, males in Somalia may be more exposed to hearing impairment due to their involvement in military or militia activities, which can expose them to loud noises, explosions, or gunshots, that can damage their hearing. Additionally, males in Somalia may be less likely to report or acknowledge their hearing difficulties, due to stigma, discrimination, or lack of awareness, which can lead to underreporting or underestimation of their disability status.

Similarly, disability prevalence and types vary by age group, with older people having higher rates and more types of disability than younger people do. This is consistent with the global trend of disability, which shows that disability increases with age, as the body and its functions decline due to natural aging processes, chronic diseases, or accumulated injuries. However, disability is not uncommon among younger people in Somalia, especially those aged 15-34 years, who account for about 30% of the total disability cases. This reflects the high burden of disability among the productive and reproductive age group in Somalia, which can have adverse effects on their education, employment, income, and social participation [18]. The main causes of disability among younger people in Somalia may include congenital or genetic conditions, infectious diseases, malnutrition, accidents, or violence, which can affect their physical, mental, or cognitive development and functioning. This indicates that disability in Somalia affects not only the older population, but also the younger generation, who need adequate support and services to realize their full potential and contribute to the development of the country. The study established that disability prevalence and types vary by location, with rural areas and IDP camps having higher rates and more types of disability than urban areas. This may be attributed to the different living conditions, environmental factors, and access to services and resources in different settings. Rural areas and IDP camps in Somalia may face greater challenges of disability due to factors such as poverty, insecurity, isolation, and lack of infrastructure, poor sanitation, and limited access to health care, education, water, food, and other basic needs, which can increase the risk and impact of disability. For example, rural areas and IDP camps may have more exposure to environmental hazards, such as droughts, floods, landslides, or wild animal attacks, which can cause injuries or diseases that can lead to disability. Moreover, rural areas and IDP camps may have less availability and quality of health care services, such as hospitals, clinics, pharmacies, or health workers, which can prevent or delay the diagnosis, treatment, prevention, or rehabilitation of disability. On the other hand, urban areas in Somalia may have lower rates and fewer types of disability due to factors such as better infrastructure, security, connectivity, and access to services and resources, which can reduce the risk and impact of disability. Urban areas may have more opportunities for education, employment, income, and social participation, which can enhance the well-being and empowerment of persons with disabilities.

One of the main findings is that Persons with Disabilities (PwDs) in Somalia face multiple barriers to access and benefit from social protection programs, such as cash transfers, food aid, or health insurance. These barriers include lack of identification documents, stigma and discrimination, physical and communication barriers, lack of information and awareness, and limited participation and representation in decision-making processes. These barriers are consistent with the evidence from other fragile and conflict-affected settings, where PwDs are often excluded or marginalized from humanitarian and development interventions [19]. Moreover, these barriers reflect the systemic and structural inequalities that PwDs face in their daily lives, which are exacerbated by the fragility and instability of the context [20,21]. Another finding is that the existing social protection programs in Somalia are not sufficiently responsive to the specific needs and preferences of PwDs, especially those with severe or multiple impairments, who require more specialized and individualized support. For instance, the cash transfer programs do not take into account the extra costs of living with a disability, such as assistive devices, medical care, or transportation, which may reduce the effectiveness and adequacy of the transfers [22]. Similarly, the food aid programs do not consider the nutritional needs or dietary restrictions of some PwDs, which may affect their health and well-being [23,24]. Furthermore, the health insurance programs do not cover the full range of services and treatments that PwDs may need, such as rehabilitation, mental health, or disability-specific care, which may limit their access and utilization of health services [25]. Another finding is that the perceived fairness of the selection criteria for social protection beneficiaries was low among both PwDs and non-PwDs. This is partly due to the lack of transparency and accountability of the targeting and delivery mechanisms, which may create opportunities for corruption, nepotism, or favoritism [26]. It is also partly due to the lack of inclusion and participation of PwDs and their representative organizations in the design and implementation of the social protection programs, which may lead to a mismatch between the eligibility criteria and the actual needs and vulnerabilities of PwDs [27,28]. Moreover, it is partly due to the lack of awareness and information among PwDs and non-PwDs about their rights and entitlements to social protection, which may result in low uptake, underreporting, or misperception of the programs [29].

**Limitations:** one of the key limitations of this study is that it used a functional approach to disability, based on the Washington Group Short Set of Questions (WGSSQ), which measures the difficulties that people face in performing basic activities, such as seeing, hearing, walking, or remembering. This approach does not capture the full complexity and diversity of disability experiences, nor the social and environmental barriers that persons with disabilities (PwDs) encounter in their daily lives.

**Interpretation:** the findings of this study reveal a high prevalence of disability and low access to social protection and disability-specific services in Somalia, especially among the most vulnerable groups and regions. Women, rural residents, older people, and those affected by conflict are more likely to have disabilities. The main barriers to accessing social protection include lack of awareness, stigma and discrimination, physical and financial constraints, and institutional and policy gaps.

**Generalizability:** this study includes 7, 212 households across various regions of Somalia, including urban and rural areas and internally displaced persons. This study can therefore be generalized to the broader Somali population. Additionally, the percentage of physical, visual and hearing impairment in this study is consistent with global disability types, thus enhancing the generalizability of the findings to similar global populations.

## Conclusion

The study indicates a need for urgent and comprehensive disability data collection that reflects the diversity, needs and barriers, reinforcing disability-inclusive policy frameworks to address the multiple dimensions of exclusion and discrimination faced by persons with disabilities in Somalia. There is also an increasing need for harmonizing and scaling up social protection benefits and services for persons with disabilities to improve access and coverage in settings similar to Somalia.

### What is known about this topic

- Social protection is a key instrument for reducing poverty and inequality and enhancing the well-being and inclusion of PwDs.

### What this study adds

- It provides insights on barriers hindering access to social protection among PwDs in Somalia.
